# Comparative proteomic analysis of extracellular matrix proteins secreted by hypertrophic scar with normal skin fibroblasts

**DOI:** 10.4103/2321-3868.130191

**Published:** 2014-04-06

**Authors:** Li Ma, Chengjun Gan, Yong Huang, Ying Wang, Gaoxing Luo, Jun Wu

**Affiliations:** 1 Chongqing Key Laboratory for Disease Proteomics, State Key Laboratory of Trauma, Burns and Combined Injury, Institute of Burn Research, Southwest Hospital, The Third Military Medical University, Chongqing, 400038 China; 2 School of Environmental Air Security and Pollution Control Engineering, Jinan University, Guangzhou, China; 3 The 421 Hospital, People’s Liberation Army, Guangzhou, China

**Keywords:** Extracellular matrix protein, fibroblast, proteomics, hypertrophic scar

## Abstract

The formation of hypertrophic scars (HSs) is a fibroproliferative disorder of abnormal wound healing. HSs usually characterize excessive proliferation of fibroblasts, abnormal deposition of extracellular matrix (ECM) during wound healing, associated with cosmetic, functional, and psychological problems. Owing to the role of ECM proteins in scar formation, we comparatively analyzed matrix proteins secreted by normal skin fibroblasts (NSFs) and HS fibroblasts (HSFs). The acetone-extracted secreted proteins were separated by sodium dodecyl sulfate-polyacrylamide gel electrophoresis (SDS-PAGE), and identified by mass spectrometry (MS). Based on Go annotation of MS data, the profiling of ECM proteins was established and scar-related proteins have been screened out. The functions of several ECM proteins identified by MS have been discussed, such as collagens I, VI, XII, fibronectin, decorin, lumican, and protein procollagen C endopeptidase enhancer 1 (PCPE-1). Among them, the MS result of PCPE-1 was supported by Western blotting that PCPE-1 from HSFs were significantly upregulated than that from NSFs. It is suggested that PCPE-1 could be a potential target for scar treatment. The exploration of scar related proteins may provide new perspectives on understanding the mechanism of scar formation and open a new way to scar treatment and prevention.

## Introduction

In general, a wound repair process occurs in almost all tissues after the exposure to any destructive stimulus and is one of the most complex biological processes. Hypertrophic scars (HSs), a special fibrosis caused by an injury to the deep dermis,[1] were formed after severe burns and trauma because of abnormal wound repair, which was caused by the disorders of skin tissue structure. The undesirable physical properties of HS tissue can be attributed to the presence of a large amount of extracellular matrix (ECM) proteins. HSs were characterized by persistent inflammation, the excessive proliferation of fibroblasts, and the abnormal deposition of ECM proteins.[2–6] HSs not only deform the appearance of patients but also severely affect the body function and the psychological health of patients. HSs often cause lifelong disability, leading to a huge global public health burden.[7] Although some progresses have been achieved in HS treatment, it still remains a daunting problem both as a clinical and basic science issue.

**Table:** 

Access this article online	
**Quick Response Code**:	**Website**: www.burnstrauma.com
	**DOI**: 10.4103/2321-3868.130191

Among various scar formation factors, fibroblasts sustained activation and proliferation in wound repair. Fibroblast activation resulted in α-smooth muscle actin (α-SMA) expression.[8] The α-SMA level could be increased by the upregulation of active transforming growth factor beta (TGF-β) 1 expression.[9] HS is principally associated with the overexpression of TGF-β1,[10–12] which also inhibits ECM degradation by downregulating matrix metalloproteinase-1 (MMP-1) and upregulating tissue inhibitors of matrix metalloproteinases (TIMPs).[13] The synthesis and secretion of excessive deposition of collagen fibrils in ECM and the lower expression of remodeling enzymes, including collagenase and MMPs, which mediates collagen degradation, is the biological basis of scar formation. The understanding mechanism of scar formation at molecular level may have a potential role in preventing and controlling scar formation, and relieving the economic burden of suffered patients.

Mass spectrometry (MS) has been widely used in the quantitative and qualitative analysis of a variety of proteins and peptides, with the rapid development in experiment methods, data analysis, and ionization techniques.[14–16] Most importantly, the introduction of the soft ionization techniques, matrix-assisted laser desorption ionization (MALDI) and electrospray ionization (ESI),[17–20] has paved the way for MS-based proteomics. Some MS-based research related to wound healing or ECM proteins has been published.[21–24]

In this paper, our objectives were to identify ECM proteins secreted by normal skin fibroblasts (NSFs) and hypertrophic scar fibroblasts (HSFs) using MS-based methods, and establish the profiling of ECM proteins. By comparison of ECM proteins from NSFs and HSFs, scar-related proteins were screened out. The exploration of scar-related proteins could open new perspectives for scar treatment and prevention.

## Materials and methods

### Human normal skin (NS) and hypertrophic scar (HS) samples

The protocols for human tissue sampling were approved by the Ethics Committee of Southwest Hospital, Chongqing, China. HS patients were selected according to the Vancouver Scar Scale ranging from 10 to 13 score.[25] All patients were informed about the purpose and procedure of this study and agreed to offer their tissue specimens with signed written consents. HS tissues were acquired from the patients undergoing orthopedic surgery at the Institute of Burn Research of the Third Military Medical University (TMMU). NS tissues were acquired from the patients in the Department of Urology, TMMU. After the removal of subcutaneous fatty tissues using a scalpel, skin samples were stored in liquid nitrogen immediately till sample preparation.

### Primary NSFs and HSFs

Fibroblasts from either NSFs or HSFs were isolated and cultured as follows: A piece of skin tissue was put in a sterile Petri dish and washed three times with phosphate buffered saline (PBS). After the epidermis was removed using a pair of sterile scissors, subcutaneous tissue was cut into 0.5 cm^3^ pieces and put into a 25 cm^2^ conical flask, then digested with 10 ml of 0.5% trypsin, and oscillated slightly 2 h at room temperature (RT). The digestion was terminated by adding of 10 ml Dulbecco’s modified Eagle’s medium (DMEM) containing 10% calf serum (Hyclone, Rockfield, IL, USA). After the suspension passed through the sterile filter, the tissue fragments were discarded. The suspension was centrifuged at 400 g/min for 10 min, and the supernatant was discarded. The suspension was washed 3 times with DMEM, and centrifuged again. The supernatant was discarded, the cells were moved to a 75 cm^2^ flask, and cultured in DMEM supplemented with 10% calf serum, 2 mM glutamine, 100 U/ml penicillin, and 100 µg/ml streptomycin in air 5% CO_2_ at 37°C. After 24 h, the culture medium was changed, while less adherent cells were removed. The cells at 90% confluence were passaged. The cells from passage 5 to 7 were used for the following experiments.

### Proteins secreted by NSFs and HSFs

When the NSFs or HSFs from passage 5 to 7 reached 30% confluence, they were washed with PBS and grown with serum-free culture for 6 days. The culture supernatants from the same number of fibroblasts were harvested, centrifuged at 3,000 rpm for 10 min at 4°C, and then precipitated with four volumes of ice-cold acetone overnight at −20°C. The acetone precipitated samples were centrifuged at 12,000 rpm for 30 min at 4°C, the supernatants were discarded, and the acetone leftover in the pellets was evaporated at RT. Finally, the extracted proteins from fibroblast culture supernatants were solved with lysis buffer, and the protein concentration was tested by Bradford (Thermo, Rockfield, IL, USA). The proteins in the lysis buffer were made into aliquots and saved at −80°C.

### In-gel digestion

The extracted proteins were separated by 10% sodium dodecyl sulfate-polyacrylamide gel electrophoresis (SDS-PAGE) with coomassie blue staining. The SDS-PAGE gel was manually cut into gel slices containing visible proteins. The gel slices were distained in 50 mM NH_4_HCO_3_ in 50% ethanol at 37°C for 30 min, then washed with 25 mM NH_4_HCO_3_ (pH 8.0), and dehydrated with acetonitrile. After being dried in a SpeedVac, the gel slices were reduced with 10 mM dithiothreitol (DTT) for 1 h at 56°C, and alkylated with 55 mM iodoacetamide containing 6 M guanidine hydrochloride in a dark room for 45 min. Then the gel slices were washed with 25 mM NH_4_HCO_3_, dehydrated with acetonitrile, and dried in a SpeedVac concentrator. The dried gel pieces were reswollen with 25 µL of 25 mM NH_4_HCO_3_ containing 0.5 µg of trypsin (modified sequencing grade; Promega, Madison, WI, USA) and 0.1% n-octyl glucoside (W/V) and digested at 37°C overnight. The peptides were extracted twice with 50 µL of 5% formic acid and 50% acetonitrile by sonication for 10 min, respectively. The combined extracts were evaporated to about 2 µL in a SpeedVac and stored at −80°C for future MS analysis.

### High performance liquid chromatography (HPLC)-chiptandem MS (MS/MS)

All nano-LC-MS/MS experiments were performed on an Agilent 6330 ion trap LC-MS/MS (Agilent Technologies, Santa, CA, USA). The system was equipped with an HPLC chip that was automatically located and positioned into the MS nanospray chamber. The chip contained a Zorbax 300 SB-C18 (43 mm × 75 µm, 5 µm) column and a Zorbax 300 SB-C18 (40 nL, 5 µm) enrichment column. The sample was loaded into the enrichment column at a flow rate of 4 µL/min with 97% solvent A (H_2_O/0.1% formic acid (FA)) and 3% solvent B (90% acetonitrile (ACN)/10% H_2_O/0.1% FA). Tryptic peptides were eluted from the reversed phase column into the mass spectrometer using a linear gradient from 3% B to 45% B in 90 min and from 45% B to 90% B in 10 min at a flow rate of 300 nL/min. The nanoelectrospray ion source was used with a spray voltage of 1.7–2.0 kV and the skimmer voltage was set at 40.0 V. The flow rate of drying gas nitrogen was 4 L/min with a gas temperature of 275°C. The fragmentation amplitude was 1.25 V. The SmartFrag was 30–200% out of 1.3 V. Positive ion mode was adopted at data-dependent mode with dynamic exclusion. Data between m/z 200 and 2,000 were recorded.

### MS data processing

MS data were searched against the International Protein Index (IPI) human database (version 3.43) using the Agilent Spectrum Mill Server software. First, peak lists were created with the Spectrum Mill Data Extractor program under the following conditions: Scans with the same precursor ±1.4 m/z were merged within a time frame of ± 15 s. Precursor ions had a minimum signal to noise value of 25 with charges up to a maximum of 5. The ^12^C peak was determined by data extractor. The Spectrum Mill MS/MS was used to search against the IPI human database for tryptic peptides with a mass tolerance of ±2.5 Da for the precursor ions and a tolerance of ±0.7 Da for the fragment ions. Cysteine carboxymethylation and methionine oxidation was used as a fixed modification and a variable modification, respectively. Two missed cleavages were allowed. The Spectrum Mill autovalidation was performed first in the protein details mode and then in the peptide mode. Minimum scores, minimum scored peak intensity, forward minus reversed score threshold, and rank 1 minus rank 2 score threshold for peptides were dependent on the assigned precursor charge. To eliminate redundancy, the protein summary mode groups of all proteins that have at least one common peptide, and only the highest scoring member of each protein group is shown and counted in the protein list.

### Gene ontology (GO) annotation

Go annotation was performed on secreted proteins. The physiological process and function of these secreted proteins were analyzed. The results demonstrated these proteins were involved in multiple physiological processes including scar formation.

### Western blotting

Equal amounts of secreted proteins from NSF and HSF culture supernatants were transferred to poly(vinylidene fluoride) (PVDF) membrane (Millipore, Billerica, MA, USA). The membrane was blocked with Tris-buffered saline (TBS) containing 5% non-fat powdered milk for 1 h at RT and then incubated with rabbit antihuman PCPE1 antibody (1:1200) at 4°C overnight. The membrane was subsequently washed with TBS containing 1% Tween 20, incubated with HRP-labeled goat anti-rabbit secondary antibody (Boster, Wuhan, China, 1:2000) for 1 h at RT, washed and visualized with electrochemiluminescent (ECL) Western Blotting Detection Reagents (Pierce, Appleton, WI, USA).

### Statistics

Statistical significance was evaluated using either a 2-tailed unpaired Student’s t test or nonparametric analysis if the SDs were significantly different between the 2 groups being tested. Throughout the text, figures, and legends, the following symbols are used to denote statistical significance: * P < 0.05; ** P < 0.01.

## Results

### GO analysis of secreted proteins from NSFs and HSFs

**Figure 1:**
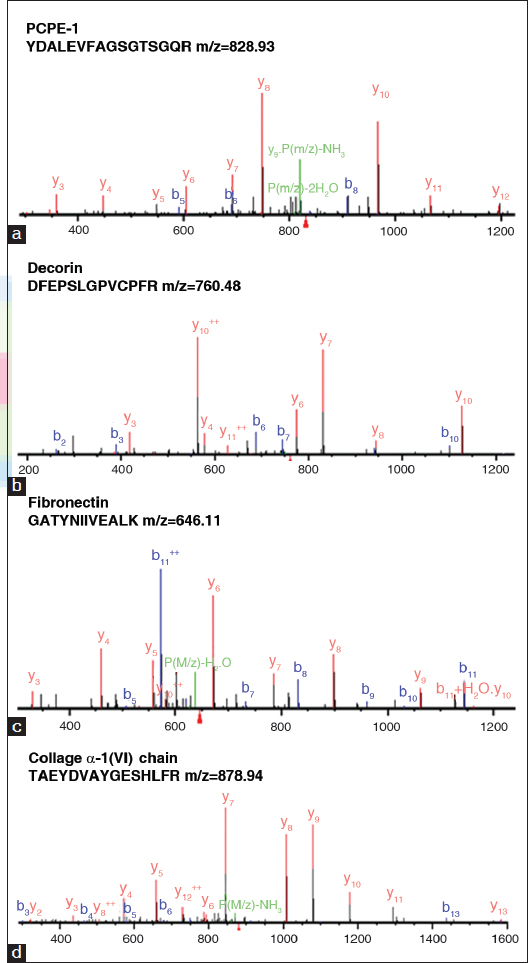
MS/MS spectra of the peptides from identified extracellular matrix (ECM) proteins corresponding to (a) the procollagen C endopeptidase enhancer 1 (PCPE-1) peptide (YDALEVFAGSGTSGQR), (b) the decorin peptide (DFEPSLGPVCPFR), (c) the fibronectin peptide (GATYNIIVEALK), and (d) the collagen α-1 (VI) chain peptide (TAEYDVAYGESHLFR).

The tryptic digested peptides were characterized by HPLC-chip-MS/MS. A total of 82 and 79 proteins were identified from the proteins secreted by NSFs and HSFs, of which 36 proteins from NSFs and 34 proteins from HSFs were annotated as ECM proteins by GO analysis, respectively. Totally, 49 proteins were identified from both cells. Among ECM proteins, 21 common proteins [Table 1] were from both cells, 13 specific proteins from HSFs [Table 2], and 15 specific proteins from NSFs [Table 3]. Several MS/MS spectra from four identified peptides have been shown in Figure 1.

**Table 1:** **Twenty-one commonly secreted proteins from NSFs and HSFs**

Protein definition	Accession number	pI	MW
Adipocyte enhancer-binding protein 1	IPI00745313	5.05	130,929
Collagen α-1(I)	IPI00297646	5.61	138,912
Collagen α-1(VI)	IPI00291136	5.26	108,530
Collagen α-3(VI)	IPI00022200	6.27	343,671
Collagen α-1(XII)	IPI00329573	5.38	333,148
Type IV collagenase	IPI00027780	5.26	73,883
Complement C1r subcomponent	IPI00296165	5.89	80,174
Complement C3	IPI00783987	6.02	187,149
EGF-containing fibulin-like extracellular matrix protein 1	IPI00029658	4.95	54,641
Fibronectin	IPI00414283	5.53	256,513
Galectin-3-binding protein	IPI00023673	5.13	65,331
Glia-derived nexin	IPI00009890	9.35	44,003
Laminin subunit α-4	IPI00329482	5.90	202,529
Laminin subunit γ-1	IPI00298281	5.01	177,608
γ-2-macroglobulin	IPI00478003	6.00	163,279
Pappalysin-1	IPI00001869	5.78	181,142
Pentraxin-related protein PTX3	IPI00029568	4.90	42,020
Plasma protease C1 inhibitor	IPI00291866	6.09	55,155
Plasminogen activator inhibitor 1	IPI00007118	6.67	45,060
Sulfhydryl oxidase 1	IPI00003590	9.13	82,578
Transforming growth factor-β-induced protein ig-h3	IPI00018219	7.62	74,681

NSF = Normal skin fibroblast, HSF = Hypertrophic scar fibroblasts, MW = Molecular weight, pI = Isoelectric point, EGF = Epidermal growth factor

**Table 2:** **Fifteen specific secreted proteins from NSFs**

Protein definition	Accesstion number	pI	MW
Antithrombin-III	IPI00032179	6.12	52,692
Apolipoprotein A-I	IPI00021841	5.56	30,778
Collagen α-2(VI) chain	IPI00304840	5.85	108,580
Complement C4-B	IPI00654875	6.74	192,795
α-fetoprotein	IPI00022443	5.48	68,678
Ficolin-3	IPI00293925	6.20	32,903
Histidine-rich glycoprotein	IPI00022371	7.09	59,579
Interleukin-1 family member 10	IPI00103482	4.95	16,943
Lumican	IPI00020986	6.16	38,429
Nidogen-1	IPI00026944	5.14	136,454
Nidogen-2	IPI00028908	5.08	151,396
Peroxidasin homolog	IPI00016112	6.79	165,276
Thyroxine-binding globulin	IPI00292946	5.87	46,325
Inter-α-trypsin inhibitor heavy chain H1	IPI00292530	6.31	101,390
Vitronectin	IPI00298971	5.55	54,306

NSF = Normal skin fibroblast, MW = Molecular weight, pI = Isoelectric point

**Table 3:** **Thirteen specific secreted proteins from HSFs**

Protein definition	Accession number	pI	MW
Cathepsin B	IPI00295741	5.88	37,822
Complement C1s subcomponent	IPI00017696	4.86	76,685
Interstitial collagenase	IPI00008561	6.47	54,007
Decorin	IPI00012119	8.75	39,747
Ectonucleotide pyrophosphatase/phosphodiesterase family member 2	IPI00303210	8.50	105,212
Fibulin 1	IPI00296534	5.11	77,262
Follistatin-related protein 1	IPI00029723	5.39	34,986
78 kDa glucose-regulated protein	IPI00003362	5.07	72,422
Pigment epithelium-derived factor	IPI00006114	5.97	46,343
Procollagen C-endopeptidase enhancer 1	IPI00299738	7.41	47,973
Stromelysin-1	IPI00027782	5.77	53,978
Tumor necrosis factor receptor superfamily member 11B	IPI00298362	8.71	46,040
WAP four-disulfide core domain protein 5	IPI00152461	8.40	24,238

HSF = Hypertrophic scar fibroblasts, MW = Molecular weight, pI = Isoelectric point

**Figure 2:**
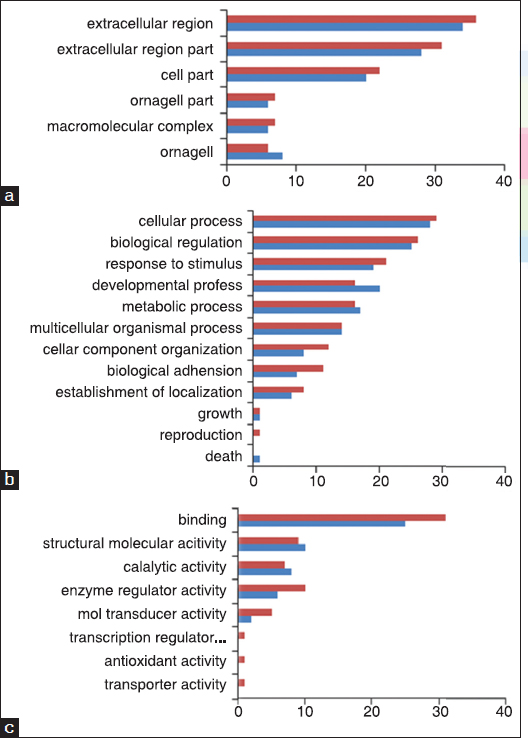
Gene ontology (GO) analysis of extracellular matrix (ECM) proteins secreted by normal skin fibroblasts (NSFs) and hypertrophic scar fibroblasts (HSFs), with (a) component annotation, (b) process annotation, and (c) activity annotation. Fibroblast culture supernatants were collected at 90% confluence between passage 5 and 7. Red bar stands for NSF and blue bar stands for HSF.

As for the components of ECM proteins, it can be seen in Figure 2a that matrix proteins are mainly from extracellular region, extracellular region part, and cell parts. A total of 13 biological processes related to ECM proteins are shown in Figure 2b. The processes mainly include cellular process and biological function, response to stimulus, developmental process, and multicellular organismal process. Figure 2c shows the functional analysis of ECM proteins. It can be seen that ECM proteins involved in binding function account for 81.8 (NSFs) and 85.3% (HSFs), respectively.

### Molecular weight (MW) and isoelectric point (pI) dependence of ECM proteins

**Figure 3:**
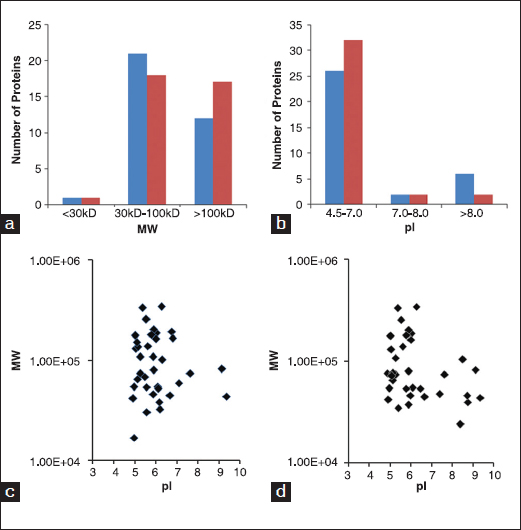
Molecular weight (MW) and isoelectric point (pI) distribution of extracellular matrix (ECM) proteins secreted by normal skin fibroblasts (NSFs) and hypertrophic scar fibroblasts (HSFs). (a) ECM proteins have been grouped into different MW bins according to their theoretical size. (b) ECM proteins have been grouped into different pI bins according to their theoretical value. Red bar stands for NSF and blue bar stands for HSF. (c and d) MW distributions of ECM proteins from NSFs and HSFS were plotted against their theoretical pIs, respectively.

The MW distribution of ECM proteins is shown in Figure 3a. Fifty and 62% of ECM proteins secreted by NSFs and HSFs have MWs in the range of 30–100 kDa, respectively. More proteins from NSFs (47%) have MW > 100 kDa than those from HSFs (35%). The pI distribution of ECM proteins over a range of 3–10 is shown in Figure 3b. Eighty-nine percent proteins from NSFs and 76% proteins from HSFs are located at pI < 7; 6% proteins from both cells at pI 7–8; fewer proteins from NSFs (6%) at pI > 8 than those from HSFs (18%). The pI distributions with MW of NSFs and HSFs are shown in Figure 3c and d.

### Verification of PCPE-1 by Western blotting

**Figure 4:**
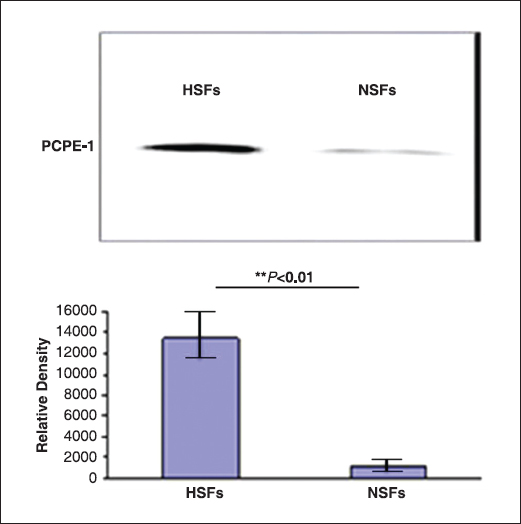
Procollagen C endopeptidase enhancer 1 (PCPE-1) expression secreted by normal skin fibroblasts (NSFs) and hypertrophic scar fibroblasts (HSFs) was validated by Western blotting (*n* = 3; * *P < 0.01).

PCEP-1 was significantly higher expressed in the culture supernatant of HSFs than in that of NSFs by Western blotting [Figure 4].

## Discussion

HS formation is a dermal fibroproliferative disorder of human dermis, leading to considerable morbidity. ECM proteins play pivotal biological regulatory roles and are the most important sources for protein therapeutics. Therefore, the analysis of ECM proteins secreted by NSFs and HSFs may provide new perspectives on HS formation and treatment. ECM contains many adhesive proteins, including fibronectin, collagen, and laminin, which generally promote cell attachment or migration.[6] Collagens stand for a large family of ECM proteins. Collagen fibril networks are stabilized by their interaction with proteoglycans and/or other collagenous and noncollagenous proteins. The synthesis ability of collagen in HSFs is obviously enhanced than that in NSFs, resulting in the excess synthesis and deposition of collagen. Excessive collagen deposition is related not only to increased collagen synthesis, but also to reduced collagen degradation. Compared pathological scars with NSs, the activity of fibroblast collagenase was significantly reduced, while the messenger ribonucleic acid (mRNA) levels of collagenase decreased significantly.[26]

It is shown from Figure 3 that the tendency of pI distribution of identified ECM proteins from NSFs and HSFs is in agreement with reported research.[27] This means that the identification of ECM proteins from fibroblasts is efficient. We identified major known ECM components, collage I and VI, laminin, fibronectin, decorin, and lumican [Figure 1], though some proteins were not detected by LC-chip-MS/MS, for example, elastin, collagen III and IV, and other proteoglycans. Some known proteins were not detected could be those reasons: The protein amount is not large enough to be identified with our instrument; some proteins are glycosylated or cross-linked, and could not be efficiently digested. For example, the formation of intermolecular covalent bonds among elastin molecules will prevent the digestion and solubilization of elastin. Additionally, more proteins would be detected if a more sensitive high quality mass spectrometer available. However, based on GO annotation, these matrix proteins are still involved 12 biological processes and eight activities.

Herein, collagens I, VI, XII and fibronectin were identified in both cell culture supernatants. Collagen VI is a non-fibrillar collagen. In collagen VI null mice, dysfunctional regulation of tendon fibrillogenesis was indirectly mediated by collagen VI.[28] Collagen XII has been classified as a fibril-associated collagen with interrupted triple helices (FACTs) and is bound to the surface of collagen I fibrils,[29] a fibril-forming protein. Collagen XII is a novel candidate marker of myofibroblasts, and/or cancer cells undergoing dedifferentiation.[30] Cancer-associated fibroblasts present a myofibroblastic phenotype, similar to the one obtained by resident fibroblasts during wound healing. Collagen XII can interact with various other ECM proteins like decorin and tenascin-X via distinct or overlapping domains.[31]

Fibronectin is a large ECM glycoprotein with a MW of 440 kDa and exists as a protein dimer. Fibronectin not only binds to membrane-spanning receptor proteins called integrins, but also binds with ECM protein such as collagen, fibrin, and syndecans.[32] Fibronectin regulates dermal fibroblast cell behavior during wound repair. The fragmentation of fibronectin by proteases has been suggested to promote wound contraction, a critical step in wound healing. Fibronectin helped the deposition of collagen in the wounded site and was observed to be closely associated with the newly deposited collagen fibrils. An *in vitro* study with native collagen has demonstrated that fibronectin bound to collagen III.[33] Fibronectin also regulated latent TGF-β1 by controlling matrix assembly of latent TGF-β1-binding protein-1.[34]

A large number of noncollagenous ECM proteins have been implicated in regulating collagen fibrillogenesis, including small leucine-rich repeat proteoglycans (SLRPs) like decorin and lumican. Lumican and decorin was founded in NSF and HSF culture supernatants, respectively. Decorin was found in HSFs consistent with the view that decorin at the messenger RNA and protein level was significantly higher in the skin lesions of patients with nephrogenic systemic fibrosis than healthy subjects.[35] Decorin is a natural anticancer agent and reduced level of decorin was associated with more aggressive disease, such as tumorigenesis, tumor invasion, and/or tumor growth.[36,37] Interestingly, lumican plays a restrictive role on prostate cancer progression, and it is postulated that lumican could be a valuable marker in prostate cancer staging.[37] Interestingly, HSFs exhibit excessive proliferation, apoptosis resistance, and atypical differentiation, which are the characteristics of malignant tumors. This maybe explained why some scar-related proteins also affect cancer progression. Because of the limitation of the used mass spectrometer and the differentiation from sample preparation, the proteins observed in HSFs or NSFs can only stand for more proteins appeared in MS, and is a primary proteomic profiling of scar-related proteins and further quantitative proteomic analysis need to be carried out.

Here, we elaborated a potential role of protein procollagen C endopeptidase enhancer 1 (PCPE-1) in HS formation. PCPE-1, a 55-kDa ECM glycoprotein without intrinsic proteolytic activity, containing two N-terminal CUB (complement-Uegf-BMP1) domains and a C-terminal NTR (netrin-like) domain, was found to be one of proteins related to fibrosis,[38,39] but no mass-based studies have reported on the relationship between PCPE-1 and skin fibrosis so far. The CUB domains of PCPE-1 can bind specifically to type I procollagen C propeptides leading to potential fibrosis, while the NTR domains of PCPE-1 may inhibit matrix-degrading proteinases.[40] PCPE-1 can enhance the activity of procollagen C proteinases (PCPs) significantly. The activity of PCPs is necessary for the conversion of soluble procollagen into the complete formation of collagen fibrils.[41] PCPs can cleave the C propeptides of procollagen I to III and the N-propeptides of procollagen V, which can cause fibrosis, and this is an important step for assembling the major fibrous components of vertebrate ECM.[39,42] During scar formation, TGF-β was highly expressed in HSFs, whereas, TGF-β can increase the PCP expression. In the process of scar formation, the higher expression of PCPE can cause the increase of procollagen into collagen fibrils; Secondly, the markable increase of PCPE-1 in HSF culture supernatants would reduce the production of matrix-degrading proteinases. All together, the increase of PCPE-1 promoted scar formation. Our MS results of PCPE-1 was supported by Western blotting [Figure 4] and that the amount of PCPE-1 from HSFs was much more than that from NSFs, whereas, the function of PCPE-1 in scar formation still needs further study. No matter what mechanism PCPE-1 has, it plays a vital role in scar formation. Thus, to control the production of PCPE-1 may provide new insights into scar treatment.[43]

## Conclusions

We established the profiling of extracellular proteins from NSFs and HSFs. A total of 36 and 34 ECM proteins were identified from NSF and HSF culture supernatants, respectively. By comparing ECM proteins from both cells, scar-related proteins were screened out, the function of several major ECM proteins were discussed in detail. PCPE-1 was found to be related to scar formation, and might be a new target for scar treatment. Some scare related ECM proteins could open new perspectives for understanding the mechanism of scar formation and provide new methods for scar treatment.
